# ACROKERATOSIS VERRUCIFORMIS OF HOPF ASSOCIATED WITH DILATED CARDIOMYOPATHY

**DOI:** 10.4103/0019-5154.55647

**Published:** 2009

**Authors:** Feroze Kaliyadan, Jayasree Manoj, S Venkitakrishnan

**Affiliations:** *From the Department of Dermatology, Amrita Institute of Medical Sciences, Kochi, Kerala - 682 026, India. E-mail: ferozkal@hotmail.com*

Sir,

A 21-year-old female patient, a known case of dilated cardiomyopathy (DCM), was referred to us from the department of cardiology for recurrent warty skin lesions, predominantly on the extremities. A biopsy confirmed the diagnosis of acrokeratosis verruciformis of Hopf. We present this case to highlight the possibility of an association between acrokeratosis verruciformis and DCM, considering the fact that a defect in the Sarco/Endoplasmic Reticulum Calcium-ATPase (*SERCA*) gene could play a role in the pathogenesis of both these conditions.

**Figure 1:**
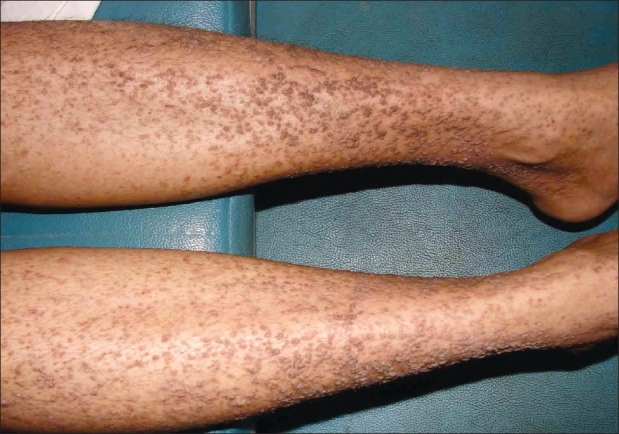
Warty papules on both lower legs

**Figure 2:**
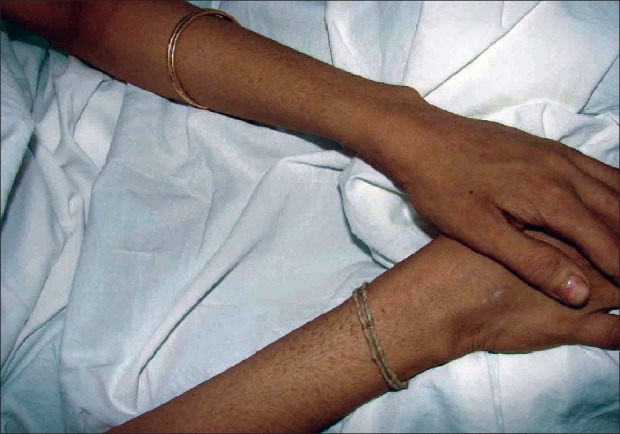
Warty papular lesions hands and forearms

**Figure 3:**
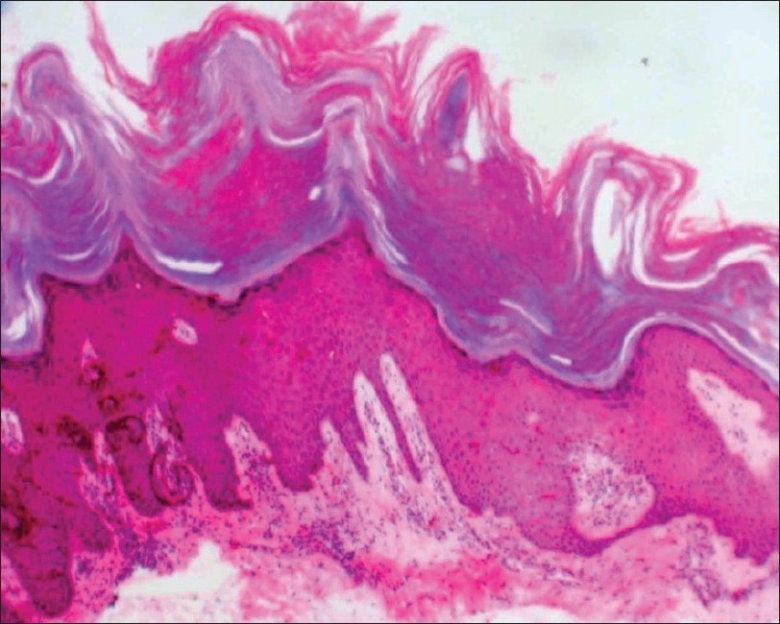
Histopathology showing typical hyperkeratosis and papillomatosis (H and E, ×40)

**Figure 4:**
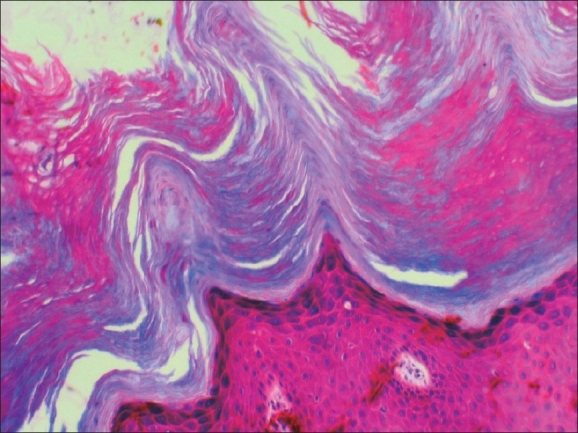
Close-up showing hyperkeratosis and papillomatosis (H and E, ×100)

Initially, the patient presented to the department of cardiology with complaints of persistent dyspnea of two months duration. She was evaluated and diagnosed to have DCM based on the results of electrocardiogram that showed sinus tachycardia with a rate of 110/min, PR interval of 0.16 seconds, right atrial enlargement was present, Q in lead II, III, aVF, poor R wave in lead V1–V3, and Q in lead V5–V6. Echocardiogram had also revealed dilated left ventricle with severe left ventricular dysfunction. Other systemic examinations were within normal limits. She was referred to the department of dermatology for warty, asymptomatic skin lesions, predominantly located over the extremities. The patient gave a history of recurrent skin lesions of one year duration. The lesions were relatively asymptomatic. There was no history of vesiculation or exacerbation in summer. The patient had not noticed any similar lesions over body flexures. There was no significant family history of any similar skin disease or any other relevant systemic disease. On clinical examination, there were extensive papular, slightly verrucous lesions, chiefly over the lower limb with scattered lesions over the forearms [Figures 1 and 2]. There were no evident pustules, vesicles, or bullous lesions. No other significant skin or mucosal lesions were seen. The possibilities of acrokeratosis verrruciformis of Hopf and Darier's disease were considered. A biopsy was taken which revealed epidermal hyperkeratosis and parakeratosis with areas showing extensive papillomatosis. No significant suprabasal acantholysis or corps-ronds were seen. There was no evidence of koilocytosis [Figures 3 and 4]. Surrounding epidermis showed mild acanthosis. There was no evidence suggestive of amyloid deposits. Congo red and PAS were negative. The histopathological features suggested a diagnosis of acrokeratosis verruciformis.

Both Darier's disease and acrokeratosis verruciformis of Hopf are considered to be allelic to each other[1,2] and are considered to be caused by separate defects in *ATPA2* gene coding for SERCA 2.[3] Coexistence of both these conditions has been documented in the past.[4] It is still not known why mutations in the widely expressed SERCA2 can manifest as a focal skin disease without any other systemic involvement. DCM is a disease which causes weakening of the heart muscle or myocardium resulting in a decrease in cardiac output. The condition progressively worsens, eventually resulting in heart failure. Calcium is one of the key substances regulating heart muscle contraction and relaxation. Following contraction, pump proteins (SERCA) actively remove calcium from the myocardial cells, and the heart muscle relaxes as the calcium levels fall. It is thought that this mechanism becomes faulty in DCM, resulting in the heart muscle being unable to relax completely between contractions. However, in general, no consistent correlation has been made between skin conditions like Darier's disease, where the same SERCA mechanism is affected, and other significant cardiac illnesses.[5,6] Interestingly, though there are isolated reports of acrokeratosis verruciformis occurring in association with DCM.[7]

One of the reasons given for the apparent localization of Darier's disease only to the skin is the presence of different subtypes of SERCA2. SERCA2a occurs chiefly in cardiac and skeletal muscles, whereas SERCA2b occurs ubiquitously and is coexpressed with the related SERCA 3 in many tissues. Both SERCA2a and SERCA2b are present in epidermis, although the latter may predominate.[8] The absence of coexpressed SERCA3 in epidermis may explain the localization of Darier's disease to the skin.[8] While the SERCA defect is implicated in the pathogenesis of acrokeratosis verruciformis, the specific mutation is likely to be different, which probably explains the absence of dyskeratosis in acrokeratosis verruciformis of Hopf. The specific defect has been linked to a heterozygous P602L mutation in *ATP2A2*. This mutation predicts a nonconservative amino acid substitution in the ATP binding domain of the molecule. Functional analysis of the P602L mutant SERCA has showed that it has lost its ability to transport Ca^2+^, thus leading to loss of function.[1] In our patient, considering the extensive lesions, there are possibilities of a overlap of Darier's and acrokeratosis, however, histopathology did not show any evidence to suggest Darier's. Could it be possible that the specific mutation in Darier's disease is restricted to the skin, while in acrokeratosis verruciformis it might have an implication of systemic involvement? Unfortunately, almost all available studies regarding SERCA-related skin and systemic studies have been restricted specifically to Darier's disease. More studies are probably warranted for studying cardiac functional defects and specific SERCA subtype expression, specifically in patients with acrokeratosis verruciformis.
